# Monocytes and Monocyte-Derived Antigen-Presenting Cells Have Distinct Gene Signatures in Experimental Model of Multiple Sclerosis

**DOI:** 10.3389/fimmu.2019.02779

**Published:** 2019-11-26

**Authors:** Kelly L. Monaghan, Wen Zheng, Gangqing Hu, Edwin C. K. Wan

**Affiliations:** ^1^Department of Microbiology, Immunology, and Cell Biology, West Virginia University, Morgantown, WV, United States; ^2^Bioinformatics Core, West Virginia University, Morgantown, WV, United States; ^3^Department of Neuroscience, West Virginia University, Morgantown, WV, United States; ^4^Rockefeller Neuroscience Institute, West Virginia University, Morgantown, WV, United States

**Keywords:** multiple sclerosis, experimental autoimmune encephalomyelitis, monocytes, antigen-presenting cells, RNA-Seq

## Abstract

Multiple sclerosis (MS) is a chronic inflammatory disease mediated by a complex interaction between the autoreactive lymphocytes and the effector myeloid cells within the central nervous system (CNS). In a murine model of MS, experimental autoimmune encephalomyelitis (EAE), Ly6C^hi^ monocytes migrate into the CNS and further differentiate into antigen-presenting cells (APCs) during disease progression. Currently, there is no information about gene signatures that can distinguish between monocytes and the monocyte-derived APCs. We developed a surface marker-based strategy to distinguish between these two cell types during the stage of EAE when the clinical symptoms were most severe, and performed transcriptome analysis to compare their gene expression. We report here that the inflammatory CNS environment substantially alters gene expression of monocytes, compared to the monocyte differentiation process within CNS. Monocytes in the CNS express genes that encode proinflammatory cytokines and chemokines, and their expression is mostly maintained when the cells differentiate. Moreover, monocyte-derived APCs express surface markers associated with both dendritic cells and macrophages, and have a significant up-regulation of genes that are critical for antigen presentation. Furthermore, we found that *Ccl17, Ccl22*, and *Ccr7* are expressed in monocyte-derived APCs but not the Ly6C^hi^ monocytes. These findings may shed light on identifying molecular signals that control monocyte differentiation and functions during EAE.

## Introduction

Multiple sclerosis (MS) is one of the most common neurological disorders among young adults. The etiology of MS is not known but it is widely accepted that it is autoimmune in nature. Current disease modifying agents (DMAs) for MS treatments reduce the rate of relapses, but these treatments do not effectively prevent disease progression (1, 2). This suggests that more effective therapeutic strategies are needed to prevent the progression of MS. Brain autopsy studies revealed that mononuclear myeloid cells are abundantly found in the active demyelinating lesions of patients with MS (3). It has also been demonstrated that mononuclear myeloid cells can directly mediate inflammation, demyelination, and axonal damage (4, 5). Consequently, these cells are an ideal target for novel MS therapies.

The role of mononuclear myeloid cells in neuroinflammation has been elucidated using experimental autoimmune encephalomyelitis, a murine model for MS research (4, 5). Under homeostatic conditions, parenchymal-resident microglia and the non-parenchymal CNS-associated macrophages, including perivascular, meningeal, and choroid plexus macrophages, are responsible for sensing pathogen invasion within the CNS. The phagocytic capability of these cells allows the clearance of pathogens, and cell debris following tissue damage. During the onset of aberrant inflammation in EAE, monocytes migrate to the CNS from the periphery and become the dominant myeloid cell type that promotes inflammation (6, 7). Monocytes are of hematopoietic origin and can be broadly divided into the classical Ly6C^hi^ CCR2^+^ CX_3_CR1^low^ and the non-classical Ly6C^low^ CCR2^−^ CX_3_CR1^hi^ types (8, 9). Classical monocytes are known to play a critical role in the pathogenesis of EAE, given that the CCR2-deficient mice do not develop the disease (10, 11). Upon arrival to the CNS, classical monocytes differentiate into antigen-presenting cells (APCs), via an unidentified signal (12). Unlike monocytes cultured *in vitro* with granulocyte-macrophage colony-stimulating factor (GM-CSF) and M-CSF, which differentiate into dendritic cells (moDCs) and macrophages (moMs), respectively, monocyte differentiation under inflammatory conditions *in vivo* is likely controlled by multiple signals (12–14). Although morphologically undistinguishable from microglia, recent studies suggest that the monocyte-derived APCs promote neuroinflammation during the course of EAE, whereas microglia protect the CNS by clearing debris (15). Therefore, identifying key molecules and pathways that potentially trigger monocyte differentiation into APCs, or distinguish these two cell types may help develop novel therapeutic strategies.

Using fluorescence activated cell sorting coupled with RNA-Seq analysis, we compared the transcriptomes of monocytes isolated from the bone marrow, and monocytes and monocyte-derived APCs from the spinal cords of mice during the peak stage of EAE when the clinical symptoms were most severe. Our primary focus was on the expression of cytokines, chemokines and their respective receptors, immunoregulatory molecules, and transcription factors. Here we report a substantial difference in gene expression profiles in the bone marrow monocytes compared to the CNS-infiltrated monocytes. In addition, CNS-infiltrated monocytes have a gene signature that is distinct from the monocyte-derived APCs. Furthermore, we propose that the expression of *Ccl17, Ccl22*, and *Ccr7* may serve as marker genes to distinguish between monocytes and the monocyte-derived APCs in the CNS.

## Materials and Methods

### Animals

Ten to twelve-week-old female mice on a C57BL/6J background were used. The mice were housed and bred under specific-pathogen-free conditions in the vivarium at West Virginia University Health Sciences Center. Mice were housed according to the Institutional Animal Care and Use Committee (IACUC) guidelines. Mice were maintained on a 12-h light/dark cycle and were fed/watered *ad libitum*. All protocols and procedures performed were approved by the IACUC of West Virginia University, protocol number 1609003850.

### Active Immunization for EAE Induction

Mice were lightly anesthetized with 2% isoflurane, then injected subcutaneously at the upper and lower back with a total of 200 μg of myelin oligodendrocyte glycoprotein (MOG) peptide emulsified in complete Freund's adjuvant (CFA) (Hooke Laboratories, Lawrence, MA, USA). Pertussis toxin (250 ng) was injected intraperitoneally at 2- and 24-h following the injection of emulsion. Clinical scores measured physical disability. A 5-point scale was used (Supplementary Figure 1).

### Isolation of Monocytes and Monocyte-Derived APCs From the Spinal Cord and the Bone Marrow

Mice were sacrificed at the peak of EAE (14–15 days post-induction). Spinal cords were isolated from the mice using the previously described ejection method (16). Briefly, the skull was removed from the mice exposing the brain. The lumbar column was then exposed and cut transversely at the lower portion just above the iliac crest. Ten milliliters of RPMI-10 medium was rapidly injected from the lumen of the spinal canal using a 22G needle. Spinal cords were digested with 1 mg/ml collagenase D and 20 mg/ml DNase I (MilliporeSigma, St. Louis, MO, USA) at 37°C for 30 min, then subjected to a discontinuous 30/70% Percoll gradient centrifugation. Cells in the interphase were collected, combined, and stained with the following antibodies: CD45-PE/Cy7 (30-F11); CD11b-FITC (M1/70); CD64-APC (X54-5/7.1); Ly6C-PE (HK1.4); and Ly6G-BV510 (1A8), all from Biolegend, San Diego, CA, USA. Dead cells were excluded by the LIVE/DEAD Fixable Near-IR stain (ThermoFisher Scientific, Waltham, MA, USA). Monocytes, characterized as CD45^+^ CD11b^+^ CD64^+^ Ly6C^hi^ Ly6G^−^, and monocyte-derived cells, characterized as CD45^+^ CD11b^+^ CD64^+^ Ly6C^low/−^ Ly6G^−^ were sorted using the BD FACSAria III sorter.

To isolate monocytes from the bone marrow of the mice, femurs and tibias were flushed with RPMI-10 medium. Monocytes were first enriched using the monocyte isolation kit (Miltenyi Biotec, Bergisch Gladbach, Germany), then stained with CD45-PE/Cy7 (30-F11), CD11b-FITC (M1/70), Ly6C-PE (HK1.4), Ly6G-BV510 (1A8), and CD11c-Percp/Cy5.5 (N418). Bone marrow monocytes characterized as CD45^+^ CD11b^+^ Ly6C^hi^ Ly6G^−^ CD11c^−^ were sorted using the BD FACSAria III sorter.

### Assessing Monocyte Differentiation During EAE

Mice were sacrificed at the priming phase (7 days post-induction) or at the peak of EAE (14–15 days post-induction). Blood (~200 μl) was collected, and red blood cells were removed by incubating with ACK lysis buffer. Cells from the spinal cord and the bone marrow were obtained as described above. Isolated cells were first incubated with anti-mouse CD16/CD32 (2.4G2, Fc block), then stained with the following antibodies to identify monocytes and monocyte-derived APCs using BD LSRFortessa: CD45-PE/Cy7 (30-F11), CD11b-FITC (M1/70), CD64-APC (X54-5/7.1), Ly6C-PE (HK1.4), Ly6G-BV510 (1A8), CCR2-BV421 (SA203G11), CD11c-Percp/Cy5.5 (N418), MHC II-BV510 (M5/114.15.2), MerTK-APC (DS5MMER), and CD24-BV421 (M1/69).

### RNA Isolation, RNA-Seq Library Preparation and Sequencing

RNA from the purified monocytes and monocyte-derived cells was isolated using RNeasy Plus Mini Kit (Qiagen, Hilden, Germany). Library preparation and sequencing were performed at the Genomics Core Facility of West Virginia University and Marshall University, respectively. RNA-Seq libraries were built using KAPA mRNA HyperPrep Kit (KAPA Biosystems, Wilmington MA, USA) with 300 ng of total RNA and 11 cycles of PCR according to manufacturer's recommendation. The libraries were quantified using Qubit Fluorometer, and the quality of the libraries was determined by Bioanalyzer High Sensitivity DNA Analysis (Agilent, Santa Clara, CA, USA). The libraries were sequenced using the HiSeq 1500 system in high output mode to generate 50 bp paired-end reads (Illumina, San Diego, CA, USA).

### Bioinformatics Analysis

The SALMON package (17) was used to estimate expression values using whole transcripts annotation from Gencode (vM22) as a reference (18). Expression level of a protein-coding gene was measured by TPM (transcripts per million) by pooling all annotated transcript isoforms of the gene with an option “-g” in SALMON. The TPM (log_2_) follows a bimodal distribution and a threshold of two separated actively expressed genes from those expressed at background level. A gene was defined as differentially expressed by GFold (19) if a “Gfold” value (log_2_) is higher than one, meaning that the probability of fold change in expression being larger than two is 0.99 with default settings. We further required DE gene to be actively expressed in at least one of the compared conditions. Heat map visualization of gene expression values were done by MeV (20).

Gene ontology enrichment analysis was done with the online DAVID bioinformatics resource 6.8 (21). Genes that were actively expressed in at least one of the analyzed cell types was included as background. Redundant GO terms output from DAVID were filtered by REVIGO (22) with default settings.

### Statistical Analyses

Statistical comparison between samples was done by student's *t*-test. ^*^P < 0.05; ^**^P < 0.01; ^***^P < 0.001. NS, not statistically different.

## Results

### Identification of Monocytes and the Monocyte-Derived APCs During EAE

**Figure 1:**
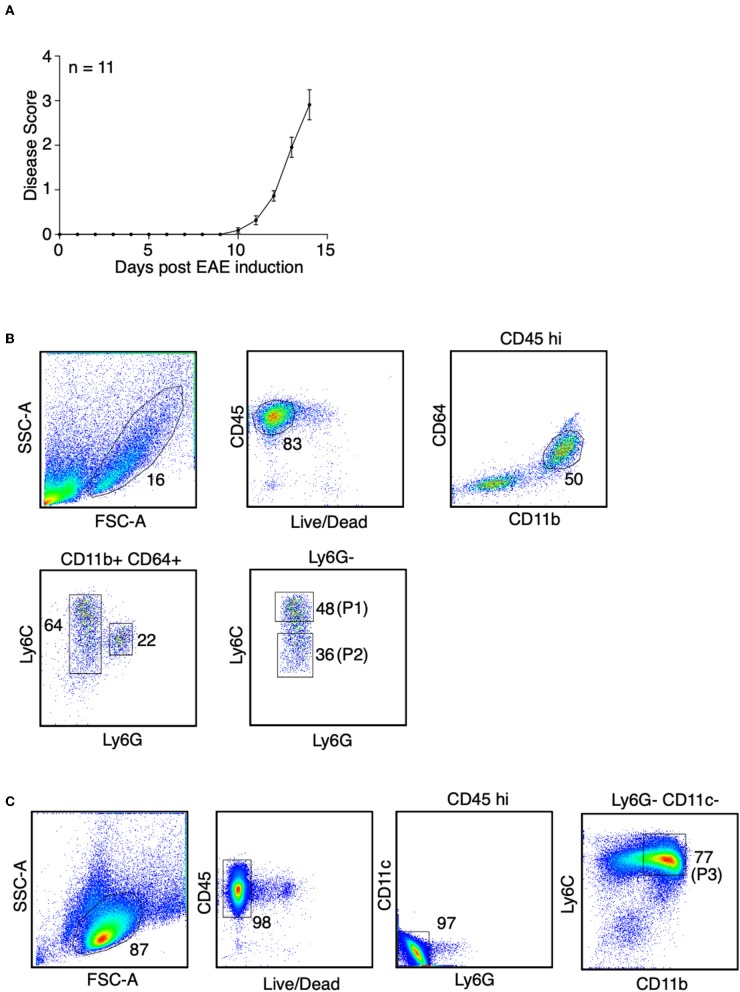
Identification of monocytes and the monocyte-derived APCs during EAE. **(A)** EAE was induced in female mice in C57BL/6J background by active immunization. Disease severity, as determined by physical disability of the mice, was measured. Shown is a combine of three independent experiments with *n* = 11. **(B)** At the peak of EAE (days 14–15), spinal cords were removed from the mice. Spinal cord monocytes (CD45^+^ CD11b^+^ CD64^+^ Ly6C^hi^ Ly6G^−^, P1) andmonocyte-derived APCs (CD45^+^ CD11b^+^ CD64^+^ Ly6C^low/−^ Ly6G^−^, P2) were purified by sorting. Shown are representative plots from three independent experiments, with a total of 18 mice. **(C)** At the peak of EAE (days 14–15), bone marrow cells were isolated from the femurs and tibias from the mice. Bone marrow monocytes (CD45^+^ CD11b^+^ Ly6C^hi^ Ly6G^−^ CD11c^−^, P3) were purified by sorting. Shown are representative plots from two independent experiments, with bone marrow cells from 13 mice were combined.

During inflammation in the CNS, monocytes and monocyte-derived APCs cannot be morphologically distinguished from microglia, non-parenchymal CNS-associated macrophages, and conventional dendritic cells (cDCs). To address this, we isolated spinal cords from the EAE-induced mice at days 14–15 post-immunization, in which the mice developed severe paralysis (score = 3, Figure 1A). Using the ejection method for spinal cord isolation we removed the leptomeninges and presumably also the non-parenchyma CNS-associated macrophages (16). Additionally, we isolated monocytes and monocyte-derived cells using antibody-based cell sorting (Figure 1B). We first gated on viable cells that highly expressed CD45, which represented the hematopoietic-derived immune cells, but not microglia. We then selected cells that expressed CD11b and CD64 (FcγRI). Selection of the CD64-positive cells excluded cDCs as CD64 is expressed in monocytes and monocyte-derived APCs, but not in cDCs (23). Since neutrophils also express CD64, the CD64-positive cells were further selected with the neutrophil marker, Ly6G. Finally, we distinguished monocytes and monocyte-derived cells by the level of Ly6C expression, since the expression of Ly6C is down-regulated during monocyte differentiation (24). Cell that expressed an intermediate level of Ly6C, which represented the partially differentiated monocytes, were excluded. Without immunization, the percentage of cells in the spinal cords that highly expressed CD45 was <0.3% (Supplementary Figures 2A,B), and among those cells the CD11b+ CD64+ monocytes and monocyte-derived cells were nearly undetectable (Supplementary Figures 2A,C). This observation indeed is reasonable because we do not expect monocyte infiltration into the CNS without immunization. To obtain a sufficient number of monocytes and monocyte-derived cells for constructing RNA-Seq libraries, sorted cells from a total of 18 mice from three independent experiments were combined.

To identify monocytes from the bone marrow during EAE, viable CD45-positive cells were selected, followed by exclusion of neutrophils and cDCs using antibodies against Ly6G and CD11c. Monocytes were defined as CD11b^hi^ Ly6C^hi^ population (Figure 1C). Sorted cells from a total of 13 mice from two independent experiments were combined.

### Monocytes and Monocyte-Derived APCs Express Signature Genes That Do Not Overlap With the CNS-Associated Macrophages and cDCs

**Figure 2:**
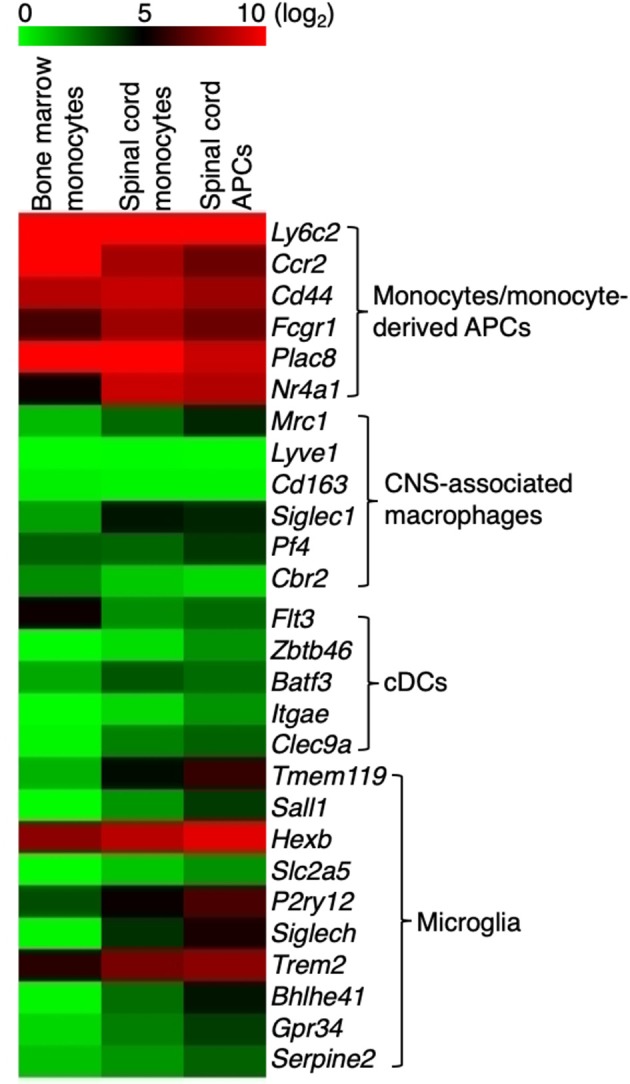
Monocytes and monocyte-derived cells express signature genes that are not overlapped by CNS-associated macrophages and cDCs. Heatmap showing the expression of signature genes in monocyte/monocyte-derived APCs, CNS-associated macrophages, cDCs, and microglia. Signature genes for each cell type were identified based on previous studies. Colors on the heatmap represent log_2_ values of TPM.

Previous transcriptome analysis studies identified signature genes that broadly characterize a mixed population of monocytes and monocyte-derived cells, CNS-associated macrophages, non-monocyte-derived cDCs, and microglia in healthy mice, during EAE, or other neuroinflammatory conditions (25–33). A recent study using single-cell RNA-Seq analysis demonstrated that cells in the CNS expressing *Ly6c2, Ccr2, Cd44*, and *Fcgr1* during EAE are likely from the monocytic origin (30). We found that these four genes were highly expressed in monocytes and the monocyte-derived APCs, together with two additional signature genes that define the monocytes/monocyte-derived cells, *Plac8* and *Nr4a1* (Figure 2). Specifically, the expression of *Cd44* distinguishes between monocyte-derived cells and microglia (31). In addition, the expression level of signature genes that identify CNS-associated macrophages (*Mrc1, Lyve1, Cd163, Siglec1, Pf4, Cbr2*) and the non-monocyte-derived cDCs (*Flt3, Zbtb46, Batf3, Itgae, Clec9a*), was either very low or absent in our population identified as monocytes or monocyte-derived APC. This suggests that our strategy for isolating monocytes and monocyte-derived cells excluded CNS-associated macrophages and cDCs (Figure 2). However, we noted that although the expression level of macroglia-associated markers *Sall1, Slc2a5, Siglech, Bhlhe41, Gpr34*, and *Serpine2* was low in monocytes and the monocyte-derived APCs, the expression of *Tmem119* and *P2ry12* was slightly increased in our monocyte-derived APC population (Figure 2). Therefore, the presence of a small number of microglia in this population of cells could not be excluded. Indeed, when we compared the expression of *Tmem119* in our monocyte-derived APC population with the gene expression profile of microglia that was previously reported (31), the estimated microglia contamination in our monocyte-derived APC population was up to 3.5% (Supplementary Figure 3). *Hexb* and *Trem2* were used previously to identify microglia but they were also highly expressed in monocytes and monocyte-derived APCs (Figure 2). *Trem2* was shown to be expressed in the inflammatory macrophages and has a detrimental role in Alzheimer's disease pathology (34). The role of *Hexb* in monocytes is not clear, but we detected that *Hexb* was highly expressed in the Ly6C^+^ monocytes isolated from the bone marrow. These findings suggested that both *Trem2* and *Hexb* are not good markers to identify microglia during EAE.

### Monocyte-Derived APCs Express Gene Signatures for Both Dendritic Cells and Macrophages

**Figure 3:**
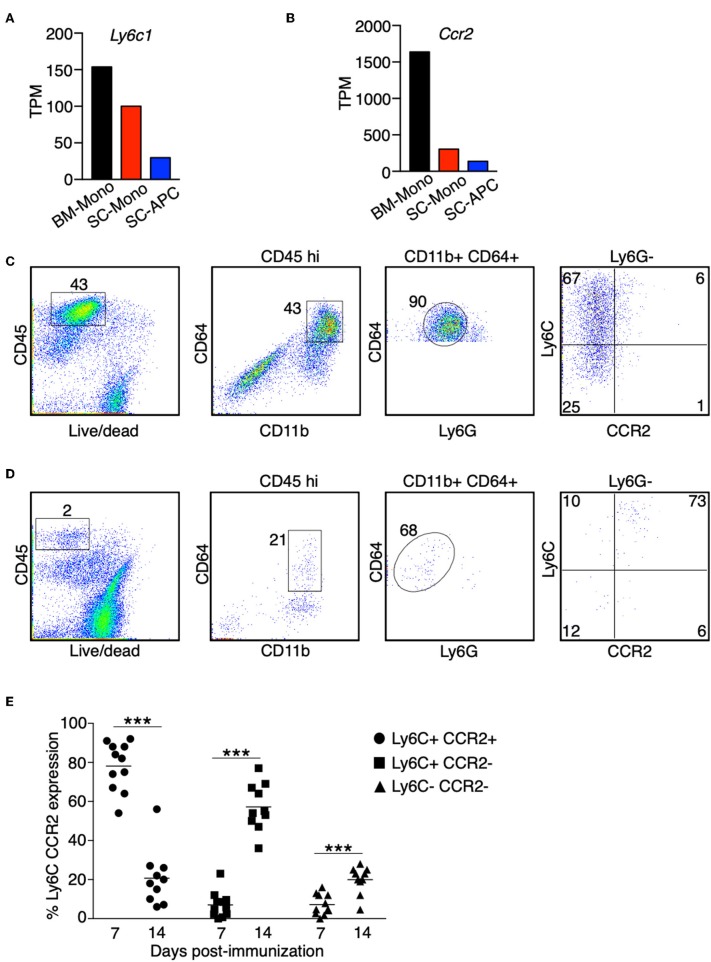
The expression of Ly6C and CCR2 in monocytes is reduced during differentiation in the CNS. **(A,B)** The gene expression level of *Ly6c1* **(A)** and *Ccr2* **(B)** in monocytes isolated from the bone marrow (BM-Mono) or spinal cords (SC-Mono), and the monocyte-derived APCs isolated from the spinal cords (SC-APC) at days 14–15 following EAE induction. Shown are TPM values from RNA-Seq analysis. **(C–E)** Spinal cord cells were isolated at days 14–15 **(C)** or day 7 **(D)** following EAE induction. The expression of Ly6C and CCR2 in the CD45^hi^ CD11b^hi^ CD64^+^ Ly6G^−^ cells was determined. Shown are representative plots from three independent experiments, with a total of 10–11 animals in each time point. **(E)** Percentage of cells expressing Ly6C and CCR2 from individual animals is shown. ***P < 0.001.

Based on the expression of surface markers, monocytes can differentiate into a heterogenous population of dendritic cells and macrophages upon stimulation with GM-CSF *in vitro* (9, 35, 36). We compared the signature genes for monocytes, dendritic cells, and macrophages in three populations of cells that we have isolated during EAE. Monocytes showed a high expression of *Ly6c* and *Ccr2* in the bone marrow. This expression was reduced when these cells migrated to the spinal cord. Expression of *Ly6c* and *Ccr2* was further reduced when these cells differentiated into APCs (Figures 3A,B). Correspondingly, we observed a reduction in the surface marker expression of Ly6C and CCR2 in the CD45^hi^ CD11b^+^ CD64^+^ Ly6G^−^ cells isolated from the spinal cord at the peak of EAE, suggesting that these cells were differentiating monocytes (Figure 3C,E). Interestingly, during the early phase of EAE when the mice did not yet develop any disease symptoms, monocytes expressed high level of Ly6C and CCR2 compared to the stage when EAE symptoms were severe (Figures 3D,E).

**Figure 4:**
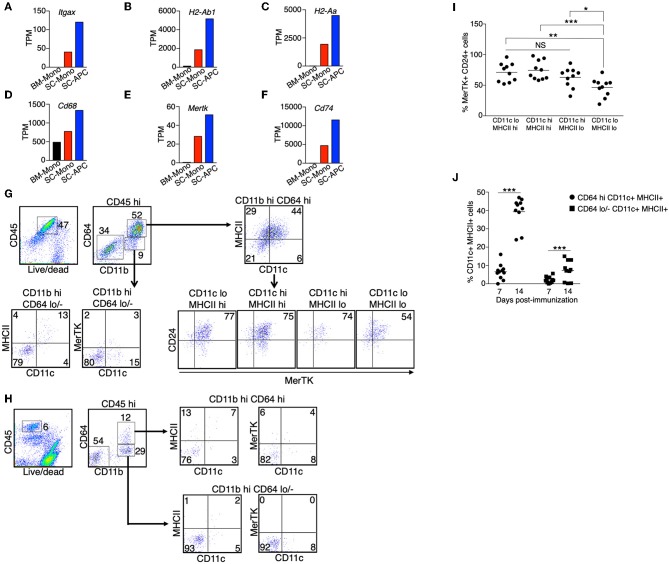
Monocyte-derived APCs expressed gene signatures for both dendritic cells and macrophages. **(A–F)** The gene expression level of *Itgax* **(A)**, *H2-Ab1* **(B)**, *H2-Aa* **(C)**, Cd68 **(D)**, Mertk **(E)**, and Cd74 **(F)** in monocytes isolated from the bone marrow (BM-Mono) or spinal cords (SC-Mono), and the monocyte-derived APCs isolated from the spinal cords (SC-APC) at days 14–15 following EAE induction. Shown are TPM values from RNA-Seq analysis. **(G–J)** Spinal cord cells were isolated at days 14–15 **(G)** or day 7 **(H)** following EAE induction. The expression of surface markers for dendritic cells (CD11c, MHC II) and macrophages (MerTK, CD24) in the CD45^hi^ CD11b^hi^ CD64^+^ Ly6G^−^ cells or the CD45^hi^ CD11b^hi^ CD64^−^ Ly6G^−^ cells was determined. Shown are representative plots from three independent experiments, with a total of 10–11 animals in each time point. **(I)** Percentage of cells expressing MerTK and CD24 within the CD11c/MHCII subpopulations from individual animals is shown. **(J)** Percentage of cells expressing CD11c and MHCII within the CD64^hi^ or CD64^lo/−^ populations from individual animals is shown. *P < 0.05; **P < 0.01; ***P < 0.001. NS, not statistically different.

In contrast to the reduced expression of signature genes for monocytes, expression of signature genes for dendritic cells (*Itgax, H2-Ab1, H2-Aa*) and macrophages (*Cd68, Mertk*) increased only during monocyte differentiation in the spinal cord (Figures 4A–E). In addition, the expression of *Cd74* was increased in the spinal cord monocytes compared to the bone marrow monocytes, and was further increased in the monocyte-derived APCs (Figure 4F). CD74 plays a critical role in antigen presentation because it mediates the assembly and trafficking of the MHC II complexes (37). Consistent with the role of CD74, recently studies demonstrated that the monocyte-derived cells have prolonged T-cell interactions compared to the CNS-resident macrophages and macroglia during EAE (30). Our results suggest that the differentiation of monocytes into APCs in the spinal cord likely increases their antigen-presenting capability.

Since we detected an increased expression of genes that are characteristic of dendritic cells and macrophages, we asked if the monocyte-derived APCs we identified from the spinal cord were a heterogenous population of dendritic cells and macrophages, or if they were a single population of cells that contain a mixed dendritic cell and macrophage phenotype. We isolated immune cells from the spinal cords at the peak of EAE (day 14–15). We used the expression CD11c and MHC II as markers for dendritic cells, and the expression MerTK and CD24 as markers for macrophages, as previously described (38). The expression of MerTK has also been recently suggested to be a marker for the ongoing differentiation of monocyte-derived macrophages during EAE (30). We found that the majority of the CD45^hi^ CD64^hi^ cells, representing cells from the monocyte origin, expressed CD11c and/or MHCII (Figure 4G). Among these cells, over 75% also expressed MerTK and CD24 (Figures 4G,I), suggesting these are cells that display both dendritic cell and macrophage phenotype. In contrast, only some of the CD45^hi^ CD64^lo/−^ cells expressed CD11c and MHC II, and these cells did not express MerTK, suggesting that they were cDCs. Intriguingly, when we analyzed the CD45^hi^ CD64^hi^ cells isolated from the spinal cord at day 7 following EAE induction, in which the mice did not start to have disease symptoms, the majority of the cells (>75%) did not express CD11c, MHC II, and MerTK (Figures 4H,J), suggesting that monocyte differentiation correlates with disease severity.

### The Inflammatory CNS Environment Substantially Induces Monocyte Activation

**Figure 5:**
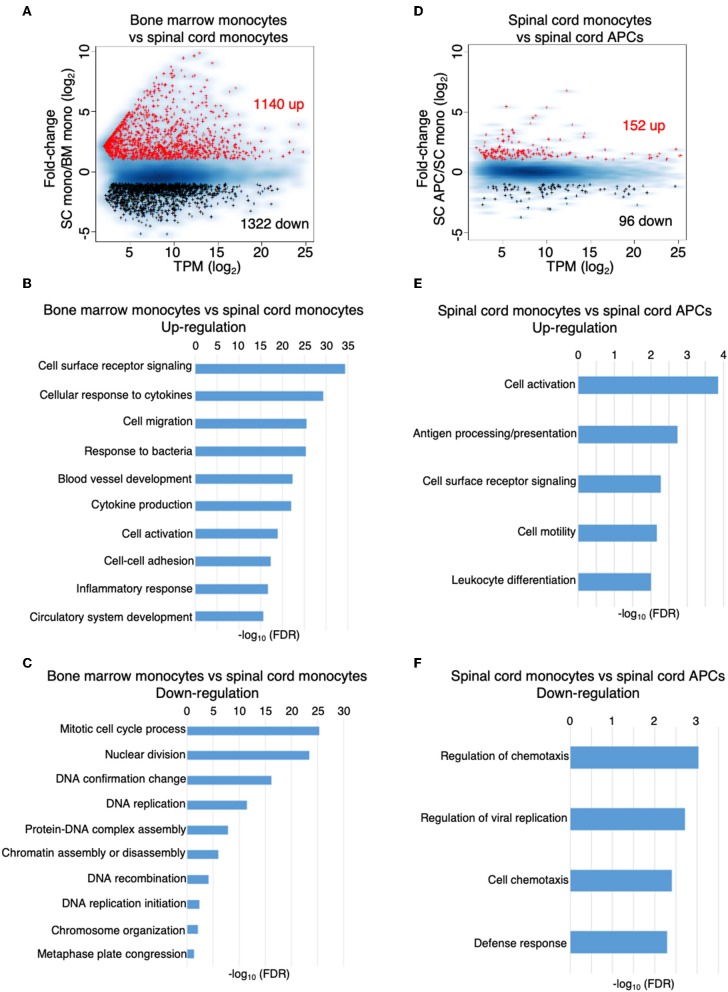
The inflammatory CNS environment substantially induces monocyte activation. **(A–C)** Comparison of the gene expression profile of the monocytes isolated from the bone marrow and the spinal cords at the peak of EAE by RNA-Seq analysis. **(A)** MA-plot of differentially expressed genes in bone marrow monocytes vs. spinal cord monocytes, with fold change ≥2 and FDR < 0.05. **(B,C)** DAVID gene ontology enrichment analysis on biological processes for genes that were up-regulated **(B)** or down-regulated **(C)** in the spinal cord monocytes compared to the bone marrow monocytes, using DAVID bioinformatics resource. **(D–F)** Comparison of the gene expression profile of the monocytes and monocyte-derived APCs isolated from the spinal cords at the peak of EAE by RNA-Seq analysis. **(D)** MA-plot of differentially expressed genes in spinal cord monocytes vs. spinal cord monocyte-derived APCs, with fold change ≥2 and FDR < 0.05**. (E,F)** DAVID gene ontology enrichment analysis on biological processes for genes that were up-regulated **(E)** or down-regulated **(F)** in the spinal cord monocyte-derived APCs compared to the spinal cord monocytes, using DAVID bioinformatics resource.

**Figure 6:**
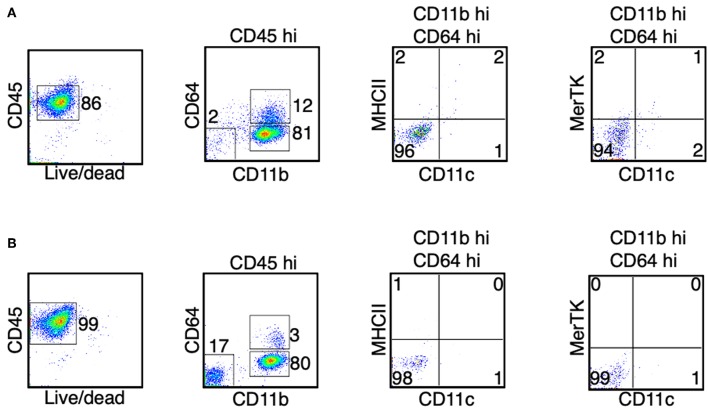
Monocytes do not differentiate in the bone marrow and the blood during the peak of EAE. **(A,B)** At the peak of EAE (days 14–15), cells from the bone marrow **(A)** and the blood **(B)** were isolated. The expression of CD11c, MHC II, and MerTK was determined in the CD45^hi^ CD11b^hi^ CD64^+^ cells. Shown are representative plots from three independent experiments, with a total of 10–11 animals in each time point.

We performed a global gene expression analysis of monocytes in the bone marrow, along with monocytes and monocyte-derived APCs, which migrated into the spinal cord during the peak of EAE. We found that the gene expression profile of monocytes was substantially changed from the bone marrow to the spinal cord, with up-regulation of 1,140 and down-regulation of 1,322 genes (fold-change > 2) (Figure 5A, Supplementary Tables 1, 2). Gene ontology enrichment analysis on biological processes revealed that the genes that were up-regulated were related to receptor signaling, cellular response to cytokines, cell migration, and cell activation (Figure 5B), whereas the genes that were down-regulated in the spinal cord were mostly related to cell division and proliferation (Figure 5C). Within the CNS, there were relatively fewer changes in gene expression when monocytes differentiated into APCs, with up-regulation of 152 and down-regulation of 96 genes (Figure 5D, Supplementary Tables 3, 4). Interestingly, the top categories of genes that were up-regulated in the monocyte-derived APCs compared to the monocytes were also related to cell activation, receptor signaling, and cell migration (Figure 5E). In addition, genes related to antigen processing and presentation, as well as cell differentiation were also up-regulated in the APCs (Figure 5E). Genes that were down-regulated during monocyte differentiation were related to chemotaxis and defense response (Figure 5F). These data suggest that cellular signals within an inflammatory CNS environment induce a significant change in monocyte function, followed by additional signals that initiate their differentiation. In support to this notion, monocyte differentiation was not observed in the bone marrow and the blood during the peak of EAE (Figures 6A,B).

### The Expression of *Ccl17, Ccl22*, and *Ccr7* Distinguishes Monocytes From Monocyte-Derived APCs

**Figure 7:**
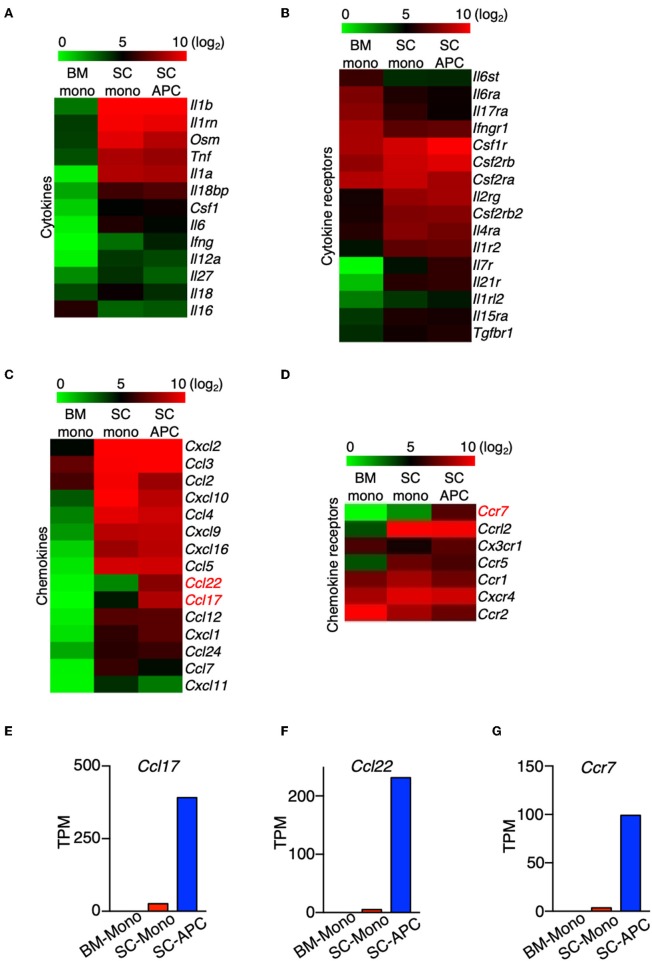
The expression of *Ccl17, Ccl22*, and *Ccr7* distinguishes monocytes from monocyte-derived APCs. **(A–D)** Heatmaps showing the differential expression of genes that encode cytokines **(A)**, cytokine receptors **(B)**, chemokines **(C)**, and chemokine receptors **(D)** from bone marrow monocytes relative to the spinal cord monocytes, and/or spinal cord monocytes relative to the spinal cord monocyte-derived APCs. Colors on the heatmap represent log_2_ values of TPM. **(E–G)** Gene expression level of *Ccl17* **(E)**, *Ccl22* **(F)**, *Ccr7* **(G)** in monocytes isolated from the bone marrow (BM-Mono) or spinal cords (SC-Mono), and the monocyte-derived APCs isolated from the spinal cords (SC-APC) at days 14–15 following EAE induction. Shown are TPM values from RNA-Seq analysis.

Monocyte migration into the CNS is critical for the pathogenesis of EAE, which is demonstrated by studies showing that mice lacking the major chemokine receptor for monocytes, CCR2, are completely resistant to the disease (10, 11). We sought to predict the pathological role of monocytes and monocyte-derived cells in the CNS by examining the expression of cytokines, chemokines, and their receptors, as well as other key immunomodulatory molecules in these cells. Compared to the monocytes in the bone marrow, the expression of *Il1a, Il1b, Il1rn, Osm*, and *Tnf* were the most significantly increased in the spinal cord monocytes and the expression was maintained during their differentiation (Figure 7A). The expression of *Il6, Il18*, and *Il18bp* was also increased, albeit to a lesser extent (Figure 7A). This result is consistent with the proinflammatory role of monocytes and the monocyte-derived cells during EAE. The expression of several cytokine receptors was also induced. Notably, the expression of *Csf1r* and *Csf2rb* was induced in monocytes and monocyte-derived APCs, and the expression *Csf2ra* was maintained at a high level in the three populations of cells that we analyzed (Figure 7B). *Csf2ra* and *Csf2rb* are subunits of the GM-CSF receptor. Several studies have confirmed a critical role of GM-CSF signaling in monocytes during EAE and MS. In addition, the receptors of several common gamma chain (γ_c_) cytokines were induced in the spinal cord monocytes and monocyte-derived APCs, including *Il2rg, Il4r, Il7r, Il15r*, and *Il21r*. This suggests that IL-4, IL-7, IL-15, and IL-21 potentially play a role in these cells. Interestingly, the expression of *Ifngr1, Il17ra, Il6ra*, and *Il6st* was reduced in the spinal cord monocytes compared to the monocytes in the bone marrow (Figure 7B).

A number of chemokine genes were up-regulated in the monocytes following migration to the CNS, with the most significant being *Ccl2, Ccl3, Ccl4, Ccl5, Cxcl2, Cxcl9, Cxcl10, Cxcl16* (Figure 7C). The expression levels of these genes were maintained in the monocyte-derived APCs, with the exception of *Ccl2* and *Cxcl10*, which were down-regulated. Importantly, the expression of *Ccl17* and *Ccl22* was absent in the bone marrow monocytes and was only expressed at low levels in the spinal cord monocytes, but the expression of these genes was significantly induced in the monocyte-derived APCs (Figures 7E,F). A recent study has shown that GM-CSF mediates CCL17 production in human monocytes and murine macrophages (39). In addition, mice lacking CCL17, CCL22, or their receptor, CCR4, develop less severe EAE (40–42). Our data suggest that *Ccl17* and *Ccl22* can distinguish between monocytes and monocyte-derived cells in the CNS during EAE. In addition, several chemokine receptor genes were up-regulated in the spinal cord during the peak of EAE, including *Ccr1, Ccr5, Ccrl2*, and *Cxcr4* (Figure 7D). Notably, similar to the expression of *Ccl17 and Ccl22, Ccr7* was minimally expressed in the bone marrow and spinal cord monocytes, but its expression was induced in monocyte-derived APCs (Figure 7G). Although *Ccr7* is also expressed in cDCs, contamination with cDCs in our APC population is unlikely as the expression of the cDC marker Zbtb46 was very low (Figure 2). Together, our data suggest that the expression of *Ccl17, Ccl22*, and *Ccr7* distinguishes between monocytes and monocyte-derived APCs in the CNS during the peak of EAE.

**Figure 8:**
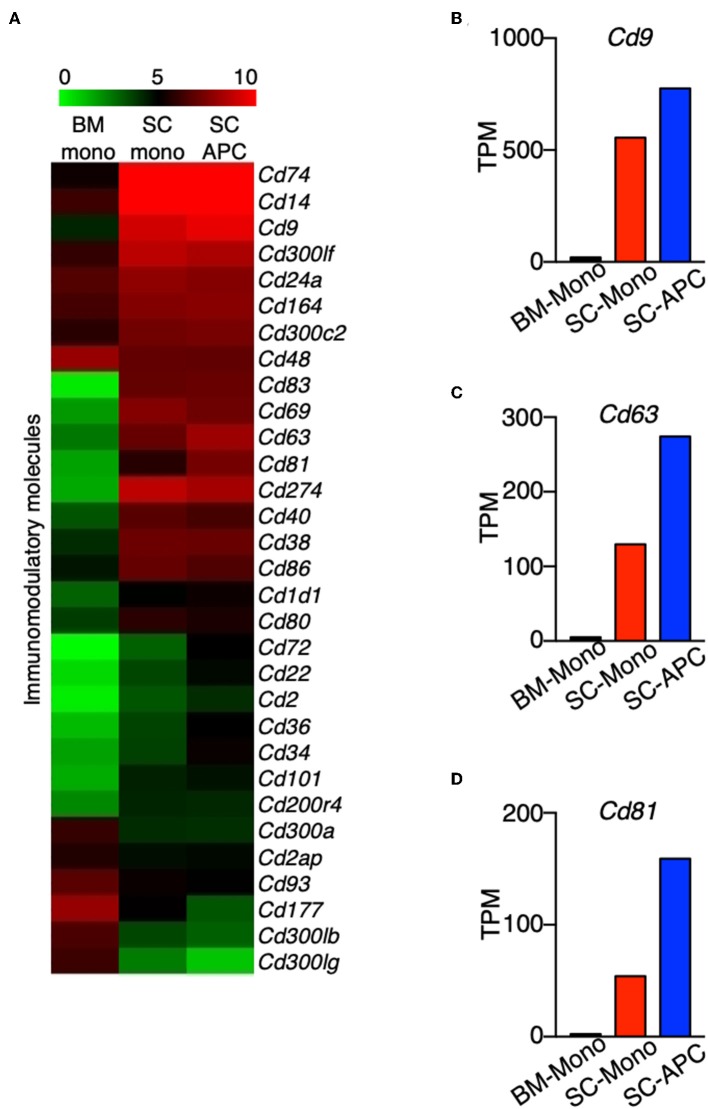
Genes encoding tetraspanins are regulated during monocyte differentiation in the CNS. **(A)** Heatmap showing the differential expression of genes that encode a selected group of immunomodulatory molecules from bone marrow monocytes relative to the spinal cord monocytes, and/or spinal cord monocytes relative to the spinal cord monocyte-derived APCs. Colors on the heatmap represent log_2_ values of TPM. **(B–D)** Gene expression level of *Cd9* **(B)**, *Cd63* **(C)**, *Cd81* **(D)** in monocytes isolated from the bone marrow (BM-Mono) or spinal cords (SC-Mono), and the monocyte-derived APCs isolated from the spinal cords (SC-APC) at days 14–15 following EAE induction. Shown are TPM values from RNA-Seq analysis.

We compared the expression of key immunomodulatory molecules that may be critical to distinguish differences in function between monocytes and monocyte-derived APCs. In both cell types, the expression of *Cd40, Cd74, Cd80*, and *Cd86* was high compared to monocytes isolated from the bone marrow (Figure 8A). This result is consistent with a recent study showing that these four genes are considered as APC signature genes (30). In addition, we found that the expression of *Cd14, Cd24a, Cd38, Cd69, Cd83, Cd164, Cd274*, and *Cd300lf* was high in the monocytes isolated from the spinal cord, and the expression was maintained in the monocyte-derived APCs (Figure 8A). Moreover, similar to the expression of *Cd74* (Figure 4F), expression of *Cd9, Cd63*, and *Cd81* was higher in the spinal cord monocytes compared to the monocytes from the bone marrow, and these genes were further up-regulated when differentiation occurred (Figures 8B–D). All three genes encode tetraspanins that were previously described to control monocyte fusion to form multinucleate giant cells (43). Taken together, our data suggest that monocytes and monocyte-derived cells commonly express several proinflammatory cytokine and chemokine genes during the peak of EAE, and the expression of genes related to antigen-presentation are further up-regulated when differentiation occurs.

### *Atf3* Is Induced in Monocytes That Migrated Into the CNS During EAE

**Figure 9:**
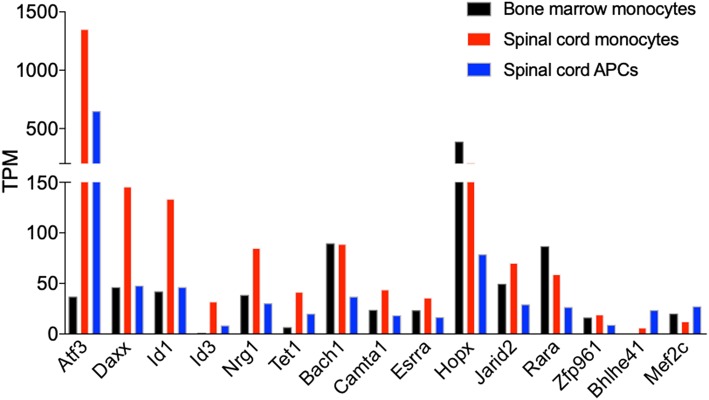
*Atf3* is induced in CNS-infiltrated monocytes during EAE. Graph shows the differential expression of genes that encode transcription factors, which are differentially expressed in monocytes relative to the monocyte-derived APCs isolated from the spinal cords at days 14–15 following EAE induction. Shown are TPM values from RNA-Seq analysis.

We identified transcription factors that had altered expression during monocyte differentiation in the CNS. The expression of 13 transcription factors was reduced during monocyte differentiation, but only two (*Bhlhe41, Mef2c*) were increased. *Atf3* was among the transcription factors that was most significantly expressed in the monocytes from the spinal cord. Its expression was reduced by half in monocyte-derived APCs (Figure 9). A recent study has shown that ATF3 promotes M2 polarization of macrophages *in vitro* (44). Whether ATF3 controls monocyte differentiation during EAE is not known. The expression of several transcription factors, including *Daxx, Id1, Id3, Nrg1*, and *Tet1*, followed the same pattern as *Atf3*, whereas the expression level of *Bach1, Camta1, Esrra, Hopx, Jarid2, Rara*, and *Zfp961* was similar in the bone marrow and spinal cord monocytes, but was reduced during monocyte differentiation. Comparing the expression of transcription factors between these cell subsets may shed light on molecular signals that control monocyte differentiation in the CNS during EAE.

## Discussion

To our knowledge, this is the first report to identify distinct gene signatures that can be used to distinguish between monocytes and monocyte-derived APCs in the CNS during inflammation. Although previous studies identified gene signatures that represent the subsets of myeloid cells in the CNS during EAE, those studies most often used the expression of *Ly6c* and *Ccr2* to identity monocytes and monocyte-derived cells as a single population of cells, and utilized the CCR2-repoter mouse strain to determine the locations and functions of these cells *in vivo*. Here we show that the expression of *Ccl17, Ccl22*, and *Ccr7* can be used to further distinguish between these cell types. Monocyte infiltration into the CNS is thought to solely promote inflammation and tissue damage during EAE. As such, a CCR2 antagonist, MK-0812, had entered a phase 2 clinical trial for treating patients with relapsing-remitting MS (NCT00239655). However, the trial was terminated and no positive results have been reported. Two recent studies have demonstrated that during the course of EAE, CNS-infiltrated monocytes differentiate first into an inducible nitric oxide synthase (iNOS)-expressing, proinflammatory state, followed by a further shift into an arginase-1 (Arg1)-expressing immune modulatory state, which may facilitate tissue repair (45, 46). This suggests that completely blocking monocyte migration into the CNS may eliminate their beneficial role in resolving inflammation. Thus, understanding the molecular signals that trigger monocyte differentiation is critical for developing immunomodulatory strategies to specifically target the deleterious effect of monocytes.

Molecular signals that control monocyte differentiation are not well-understood but are believed to be multifactorial and likely include general inflammatory mediators and those mediators which are tissue-specific. In this regard, comparison of our data with other published gene expression studies of the Ly6C^+^ cells isolated from various inflammatory conditions may provide insight on the tissue-specific monocyte differentiation. We showed that the expression of *Csf1r* and *Csf2r* was high in the monocytes and further increased following their differentiation. CSF1 receptor signaling is critical for the tissue non-specific differentiation of monocytes under various inflammatory conditions and is defined by the expression of CD11c and MHC II. GM-CSF, which acts through CSF2 receptor, is dispensable for the production of iNOS and TNF-α in monocytes (47). However, several lines of evidence support the notion that GM-CSF signaling in monocytes is critical for CNS pathogenicity during EAE (48, 49). The combined impact of CSF1 and CSF2 receptor signaling on monocyte differentiation and function requires further investigation. Moreover, the expression of several γc family receptors were detected in the monocytes and monocyte-derived APCs in the CNS, including receptor for IL-4, IL-7, IL-15, and IL-21. The combination of GM-CSF and IL-4 is known to induce the differentiation of monocytes into dendritic cells *in vitro* (50, 51). In addition, GM-CSF-induced monocyte differentiation into dendritic cells is enhanced by IL-15 but inhibited by IL-21 (52). The role of these γ_c_ cytokines and their cellular source during EAE remains to be elucidated.

Our findings suggest that *Ccl17* and *Ccl22* may serve as signature genes for the monocyte-derived APCs. These chemokines were also demonstrated to promote EAE pathology (41). Mice lacking CCL17, CCL22, or their receptor, CCR4, are partially resistant to the development of EAE (40, 42, 53). These chemokines seem to promote EAE by enhancing myeloid cell migration and their production of TNF (40, 53). GM-CSF was found to be the main stimulant for the production of CCL17 in monocytes (39). Indeed, we found that GM-CSF induces the expression of CCL17 and CCL22 in monocytes, which is dependent on the activation of STAT5 tetramerization (Monaghan et al., manuscript in preparation). Future research can explore the possibility of targeting CCL17 and CCL22 for treating EAE and MS.

An important question that remains to be addressed is whether all monocyte-derived APCs defined by our gating strategy express *Ccl17, Ccl22*, and *Ccr7*, given that this population of cells may be heterogenous in nature. One possibility is that some cells within the monocyte-derived APC population express high levels of these genes, whereas other cells do not. In this regard, single-cell gene expression analysis or the use of reporter mice (e.g., *Ccl17*-reporter mice) may provide further insight.

Our RNA-Seq analysis has shown that monocytes, once migrated to the CNS and prior cell differentiation, highly expressed proinflammatory cytokines, such as *Il1b, Osm*, and *Tnf*, and chemokines, such as *Cxcl2* and *Cxcl10* that have been shown to promote the pathogenesis of EAE (54, 55). The expression of genes that are critical for antigen presentation, such as *H2-Ab1, H2-Aa, Cd74* were significantly induced in the differentiated APCs. This suggests that differentiation of monocytes increases their antigen-presentation capability, which is known to be a critical and distinctive function of the monocyte-derived APCs compared to the CNS-resident macrophages and macroglia (30). Based on surface marker expression, these cells co-expressed markers for dendritic cells and macrophages, although cells that only expressed markers of either cell type could not be excluded.

## Conclusion

In summary, we have shown that monocytes and monocyte-derived APCs in the CNS have distinct gene expression profiles during EAE, and found that expression of *Ccl17, Ccl22*, and *Ccr7* can be used to distinguish between monocyte-derived APCs and the undifferentiated monocytes. In the past, the monocyte-derived APCs are often described in the literature as monocyte-derived macrophages, monocytes/macrophages, or simply monocyte-derived myeloid cells. We propose that this population of cells can be better named as monocyte-derived inflammatory APCs.

## Data Availability Statement

The datasets generated for this study can be found in the RNA-Seq data deposited in GEO, under the accession number GSE137801, https://www.ncbi.nlm.nih.gov/geo/query/acc.cgi?acc=GSE137801. The data that support the findings of this study are available from the corresponding author upon reasonable request.

## Ethics Statement

The animal study was reviewed and approved by IACUC of West Virginia University, protocol number 1609003850.

## Author Contributions

KM and EW designed and performed experiments, analyzed data, and wrote the manuscript. WZ performed experiments. GH designed and implemented RNA-Seq data analysis. EW supervised the project. All authors approved the final manuscript.

### Conflict of Interest

The authors declare that the research was conducted in the absence of any commercial or financial relationships that could be construed as a potential conflict of interest.

## Supplementary Material

Supplementary Figure 1 Disease symptoms and criteria for determining the clinical scores of EAE.

Supplementary Figure 2 CD64^+^ monocytes are not detected in mice without EAE induction. **(A,B)** Spinal cords from the naïve C57BL/6J mice were removed. Percentage of the CD45^+^ CD11b^+^ CD64^+^ and the CD45^+^ CD11b^+^ CD64^lo/−^ cells was determined. **(A)** Shown are representative plots from two independent experiments with a total of six mice. **(B)** Data from individual animals are shown.

Supplementary Figure 3 Comparison of the expression levels of *Tmem119, Ccl17, Ccl22*, and *Ccr7* in the monocytes and the monocyte-derived APCs to microglia. Shown are TPM values normalized with the expression of *Actb* across datasets. Percentage of microglia contamination is calculated by the expression of *Tmem119* in the microglia over spinal cord monocytes or spinal cord APCs. Gene expression data of microglia were obtained from results published previously (31).

Supplementary Table 1 A subset of genes that were up-regulated in the spinal cord monocytes compared to the bone marrow monocytes.

Supplementary Table 2 A subset of genes that were down-regulated in the spinal cord monocytes compared to the bone marrow monocytes.

Supplementary Table 3 A subset of genes that were up-regulated in the spinal cord APCs compared to the spinal cord monocytes.

Supplementary Table 4 A subset of genes that were down-regulated in the spinal cord APCs compared to the spinal cord monocytes.

## References

[1] Ransohoff RM Hafler DA Lucchinetti CF Multiple sclerosis-a quiet revolution. Nat Rev Neurol. (2015) 11:134–42. 10.1038/nrneurol.2015.14 25686758 PMC4556342

[2] Wagner CA Goverman JM. Novel insights and therapeutics in multiple sclerosis. F1000Res. (2015) 4:517. 10.12688/f1000research.6378.1 26339480 PMC4544373

[3] De Groot CJ Bergers E Kamphorst W Ravid R Polman CH Barkhof F. Post-mortem MRI-guided sampling of multiple sclerosis brain lesions: increased yield of active demyelinating and (p)reactive lesions. Brain. (2001) 124:1635–45. 10.1093/brain/124.8.1635 11459754

[4] Segal B.M. Modulation of the innate immune system: a future approach to the treatment of neurological disease. Clin Immunol. (2018) 189:1–3. 10.1016/j.clim.2018.03.003 29628125

[5] Mishra MK Yong VW. Myeloid cells - targets of medication in multiple sclerosis. Nat Rev Neurol. (2016) 12:539–51. 10.1038/nrneurol.2016.110 27514287

[6] Kierdorf K Masuda T Jordao MJC Prinz M. Macrophages at CNS interfaces: ontogeny and function in health and disease. Nat Rev Neurosci. (2019) 20:547–62. 10.1038/s41583-019-0201-x 31358892

[7] Prinz M Erny D Hagemeyer N Ontogeny and homeostasis of CNS myeloid cells. Nat Immunol. (2017) 18:385–92. 10.1038/ni.3703 28323268

[8] Ginhoux F Jung S. Monocytes and macrophages: developmental pathways and tissue homeostasis. Nat Rev Immunol. (2014) 14:392–404. 10.1038/nri3671 24854589

[9] Guilliams M Mildner A Yona S. Developmental and functional heterogeneity of monocytes. Immunity. (2018) 49:595–613. 10.1016/j.immuni.2018.10.005 30332628

[10] Fife BT Huffnagle GB Kuziel WA Karpus WJ. CC chemokine receptor 2 is critical for induction of experimental autoimmune encephalomyelitis. J Exp Med. (2000) 192:899–905. 10.1084/jem.192.6.899 10993920 PMC2193286

[11] Izikson L Klein RS Charo IF Weiner HL Luster AD. Resistance to experimental autoimmune encephalomyelitis in mice lacking the CC chemokine receptor (CCR)2. J Exp Med. (2000) 192:1075–80. 10.1084/jem.192.7.1075 11015448 PMC2193310

[12] Jakubzick CV Randolph GJ Henson PM. Monocyte differentiation and antigen-presenting functions. Nat Rev Immunol. (2017) 17:349–62. 10.1038/nri.2017.28 28436425

[13] Ushach I Zlotnik A. Biological role of granulocyte macrophage colony-stimulating factor (GM-CSF) and macrophage colony-stimulating factor (M-CSF) on cells of the myeloid lineage. J Leukoc Biol. (2016) 100:481–9. 10.1189/jlb.3RU0316-144R 27354413 PMC4982611

[14] Lacey DC Achuthan A Fleetwood AJ Dinh H Roiniotis J Scholz G.M. Defining GM-CSF- and macrophage-CSF-dependent macrophage responses by *in vitro* models. J Immunol. (2012) 188:5752–65. 10.4049/jimmunol.1103426 22547697

[15] Yamasaki R Lu H Butovsky O Ohno N Rietsch AM Cialic R. Differential roles of microglia and monocytes in the inflamed central nervous system. J Exp Med. (2014) 211:1533–49. 10.1084/jem.20132477 25002752 PMC4113947

[16] Kennedy HS Jones C 3rd Caplazi P. Comparison of standard laminectomy with an optimized ejection method for the removal of spinal cords from rats and mice. J Histotechnol. (2013) 36:86–91. 10.1179/014788813X13756994210382 24039319 PMC3770983

[17] Patro R Duggal G Love M.I Irizarry RA Kingsford C. Salmon provides fast and bias-aware quantification of transcript expression. Nat Methods. (2017) 14:417–9. 10.1038/nmeth.4197 28263959 PMC5600148

[18] Frankish A Diekhans M Ferreira AM Johnson R Jungreis I Loveland J. GENCODE reference annotation for the human and mouse genomes. Nucleic Acids Res. (2019) 47:D766–73. 10.1093/nar/gky955 30357393 PMC6323946

[19] Feng J Meyer CA Wang Q Liu JS, Shirley Liu X, Zhang Y. GFOLD: a generalized fold change for ranking differentially expressed genes from RNA-seq data. Bioinformatics. (2012) 28:2782–8. 10.1093/bioinformatics/bts515 22923299

[20] Howe EA Sinha R Schlauch D Quackenbush J. RNA-Seq analysis in MeV. Bioinformatics. (2011) 27:3209–10. 10.1093/bioinformatics/btr490 21976420 PMC3208390

[21] Jiao X Sherman BT Huang da W Stephens R Baseler M.W Lane H.C. DAVID-WS: a stateful web service to facilitate gene/protein list analysis. Bioinformatics. (2012) 28:1805–6. 10.1093/bioinformatics/bts251 22543366 PMC3381967

[22] Supek F Bosnjak M Skunca N Smuc T. REVIGO summarizes and visualizes long lists of gene ontology terms. PLoS ONE. (2011) 6:e21800. 10.1371/journal.pone.0021800 21789182 PMC3138752

[23] Langlet C Tamoutounour S Henri S Luche H Ardouin L Gregoire C. CD64 expression distinguishes monocyte-derived and conventional dendritic cells and reveals their distinct role during intramuscular immunization. J Immunol. (2012) 188:1751–60. 10.4049/jimmunol.1102744 22262658

[24] Rogers PB Driessnack MG Hiltbold Schwartz E. Analysis of the developmental stages, kinetics, and phenotypes exhibited by myeloid cells driven by GM-CSF *in vitro*. PLoS ONE. (2017) 12:e0181985. 10.1371/journal.pone.0181985 28750033 PMC5531556

[25] Bennett ML Bennett FC Liddelow SA Ajami B Zamanian JL Fernhoff NB. New tools for studying microglia in the mouse and human CNS. Proc Natl Acad Sci USA. (2016) 113:E1738–46. 10.1073/pnas.1525528113 26884166 PMC4812770

[26] Butovsky O Jedrychowski MP Moore CS Cialic R Lanser AJ Gabriely G. Identification of a unique TGF-beta-dependent molecular and functional signature in microglia. Nat Neurosci. (2014) 17:131–43. 10.1038/nn.3599 24316888 PMC4066672

[27] Buttgereit A Lelios I Yu X Vrohlings M Krakoski NR Gautier EL. Sall1 is a transcriptional regulator defining microglia identity and function. Nat Immunol. (2016) 17:1397–406. 10.1038/ni.3585 27776109

[28] Gautier EL Shay T Miller J Greter M Jakubzick C Ivanov S. Gene-expression profiles and transcriptional regulatory pathways that underlie the identity and diversity of mouse tissue macrophages. Nat Immunol. (2012) 13:1118–28. 10.1038/ni.2419 23023392 PMC3558276

[29] Goldmann T Wieghofer P Jordao MJ Prutek F Hagemeyer N Frenzel K. Origin, fate and dynamics of macrophages at central nervous system interfaces. Nat Immunol. (2016) 17:797–805. 10.1038/ni.3423 27135602 PMC4968048

[30] Jordao MJC Sankowski R Brendecke SM Sagar Locatelli G Tai YH. Single-cell profiling identifies myeloid cell subsets with distinct fates during neuroinflammation. Science. (2019) 363:eaat7554. 10.1126/science.aat7554 30679343

[31] Lewis ND Hill JD Juchem KW Stefanopoulos DE Modis LK. RNA sequencing of microglia and monocyte-derived macrophages from mice with experimental autoimmune encephalomyelitis illustrates a changing phenotype with disease course. J Neuroimmunol. (2014) 277:26–38. 10.1016/j.jneuroim.2014.09.014 25270668

[32] Li Q Lan X Han X Wang J. Expression of Tmem119/Sall1 and Ccr2/CD69 in FACS-sorted microglia- and monocyte/macrophage-enriched cell populations after intracerebral hemorrhage. Front Cell Neurosci. (2018) 12:520. 10.3389/fncel.2018.00520 30687011 PMC6333739

[33] Zeisel A Munoz-Manchado AB Codeluppi S Lonnerberg P La Manno G Jureus A. Brain structure. Cell types in the mouse cortex and hippocampus revealed by single-cell RNA-seq. Science. (2015) 347:1138–42. 10.1126/science.aaa1934 25700174

[34] Jay TR Miller CM Cheng PJ Graham LC Bemiller S Broihier ML. TREM2 deficiency eliminates TREM2+ inflammatory macrophages and ameliorates pathology in Alzheimer's disease mouse models. J Exp Med. (2015) 212:287–95. 10.1084/jem.20142322 25732305 PMC4354365

[35] Helft J Bottcher J Chakravarty P Zelenay S Huotari J Schraml BU. GM-CSF mouse bone marrow cultures comprise a heterogeneous population of CD11c(+)MHCII(+) macrophages and dendritic cells. Immunity. (2015) 42:1197–211. 10.1016/j.immuni.2015.05.018 26084029

[36] Menezes S Melandri D Anselmi G Perchet T Loschko J Dubrot J The heterogeneity of Ly6C(hi) monocytes controls their differentiation into iNOS(+) macrophages or monocyte-derived dendritic cells. Immunity. (2016) 45:1205–18. 10.1016/j.immuni.2016.12.001 28002729 PMC5196026

[37] Schroder B. The multifaceted roles of the invariant chain CD74– more than just a chaperone. Biochim Biophys Acta. (2016) 1863:1269–81. 10.1016/j.bbamcr.2016.03.026 27033518

[38] Yu YR O'Koren EG Hotten DF Kan MJ Kopin D Nelson ER. A protocol for the comprehensive flow cytometric analysis of immune cells in normal and inflamed murine non-lymphoid tissues. PLoS ONE. (2016) 11:e0150606. 10.1371/journal.pone.0150606 26938654 PMC4777539

[39] Achuthan A Cook AD Lee MC Saleh R Khiew HW Chang MW. Granulocyte macrophage colony-stimulating factor induces CCL17 production via IRF4 to mediate inflammation. J Clin Invest. (2016) 126:3453–66. 10.1172/JCI87828 27525438 PMC5004969

[40] Ruland C Renken H Kuzmanov I Fattahi Mehr A Schwarte K Cerina M. Chemokine CCL17 is expressed by dendritic cells in the CNS during experimental autoimmune encephalomyelitis and promotes pathogenesis of disease. Brain Behav Immun. (2017) 66:382–93. 10.1016/j.bbi.2017.06.010 28642092

[41] Scheu S Ali S Ruland C Arolt V Alferink J. The C-C chemokines CCL17 and CCL22 and their receptor CCR4 in CNS autoimmunity. Int J Mol Sci. (2017) 18:E2306. 10.3390/ijms18112306 29099057 PMC5713275

[42] Poppensieker K Otte DM Schurmann B Limmer A Dresing P Drews E. CC chemokine receptor 4 is required for experimental autoimmune encephalomyelitis by regulating GM-CSF and IL-23 production in dendritic cells. Proc Natl Acad Sci USA. (2012) 109:3897–902. 10.1073/pnas.1114153109 22355103 PMC3309768

[43] Champion TC Partridge LJ Ong SM Malleret B Wong SC Monk PN. Monocyte subsets have distinct patterns of tetraspanin expression and different capacities to form multinucleate giant cells. Front Immunol. (2018) 9:1247. 10.3389/fimmu.2018.01247 29937768 PMC6002745

[44] Sha H Zhang D Zhang Y Wen Y Wang Y. ATF3 promotes migration and M1/M2 polarization of macrophages by activating tenascinC via Wnt/betacatenin pathway. Mol Med Rep. (2017) 16:3641–7. 10.3892/mmr.2017.6992 28714032

[45] Giles DA Washnock-Schmid JM Duncker PC Dahlawi S Ponath G Pitt D. Myeloid cell plasticity in the evolution of central nervous system autoimmunity. Ann Neurol. (2018) 83:131–41. 10.1002/ana.25128 29283442 PMC5876132

[46] Locatelli G Theodorou D Kendirli A Jordao MJC Staszewski O Phulphagar K. Mononuclear phagocytes locally specify and adapt their phenotype in a multiple sclerosis model. Nat Neurosci. (2018) 21:1196–208. 10.1038/s41593-018-0212-3 30127427

[47] Greter M Helft J Chow A Hashimoto D Mortha A Agudo-Cantero J. GM-CSF controls nonlymphoid tissue dendritic cell homeostasis but is dispensable for the differentiation of inflammatory dendritic cells. Immunity. (2012) 36:1031–46. 10.1016/j.immuni.2012.03.027 22749353 PMC3498051

[48] Croxford AL Lanzinger M Hartmann FJ Schreiner B Mair F Pelczar P. The cytokine GM-CSF drives the inflammatory signature of CCR2+ monocytes and licenses autoimmunity. Immunity. (2015) 43:502–14. 10.1016/j.immuni.2015.08.010 26341401

[49] Spath S Komuczki J Hermann M Pelczar P Mair F Schreiner B. Dysregulation of the cytokine GM-CSF induces spontaneous phagocyte invasion and immunopathology in the central nervous system. Immunity. (2017) 46:245–60. 10.1016/j.immuni.2017.01.007 28228281

[50] Sander J Schmidt SV Cirovic B McGovern N Papantonopoulou O Hardt AL. Cellular differentiation of human monocytes is regulated by time-dependent interleukin-4 signaling and the transcriptional regulator NCOR2. Immunity. (2017) 47:1051–66.e1012. 10.1016/j.immuni.2017.11.024 29262348 PMC5772172

[51] Sallusto F Lanzavecchia A. Efficient presentation of soluble antigen by cultured human dendritic cells is maintained by granulocyte/macrophage colony-stimulating factor plus interleukin 4 and downregulated by tumor necrosis factor alpha. J Exp Med. (1994) 179:1109–18. 10.1084/jem.179.4.1109 8145033 PMC2191432

[52] Brandt K Bulfone-Paus S Foster DC Ruckert R. Interleukin-21 inhibits dendritic cell activation and maturation. Blood. (2003) 102:4090–8. 10.1182/blood-2003-03-0669 12893770

[53] Dogan RN Long N Forde E Dennis K Kohm AP Miller SD. CCL22 regulates experimental autoimmune encephalomyelitis by controlling inflammatory macrophage accumulation and effector function. J Leukoc Biol. (2011) 89:93–104. 10.1189/jlb.0810442 20940325 PMC3004518

[54] Fife BT Kennedy KJ Paniagua MC Lukacs NW Kunkel SL Luster AD. CXCL10 (IFN-gamma-inducible protein-10) control of encephalitogenic CD4+ T cell accumulation in the central nervous system during experimental autoimmune encephalomyelitis. J Immunol. (2001) 166:7617–24. 10.4049/jimmunol.166.12.7617 11390519

[55] Stoolman JS Duncker PC Huber AK Giles DA Washnock-Schmid JM Soulika AM. An IFNgamma/CXCL2 regulatory pathway determines lesion localization during EAE. J Neuroinflammation. (2018) 15:208. 10.1186/s12974-018-1237-y 30012158 PMC6048869

