# Supplementary figures and images for: Monocytes and Monocyte-Derived Antigen-Presenting Cells Have Distinct Gene Signatures in Experimental Model of Multiple Sclerosis
Source: Front Immunol. 2019 Nov 26;10:2779. doi: 10.3389/fimmu.2019.02779 (PMC6889845; doi:10.3389/fimmu.2019.02779)

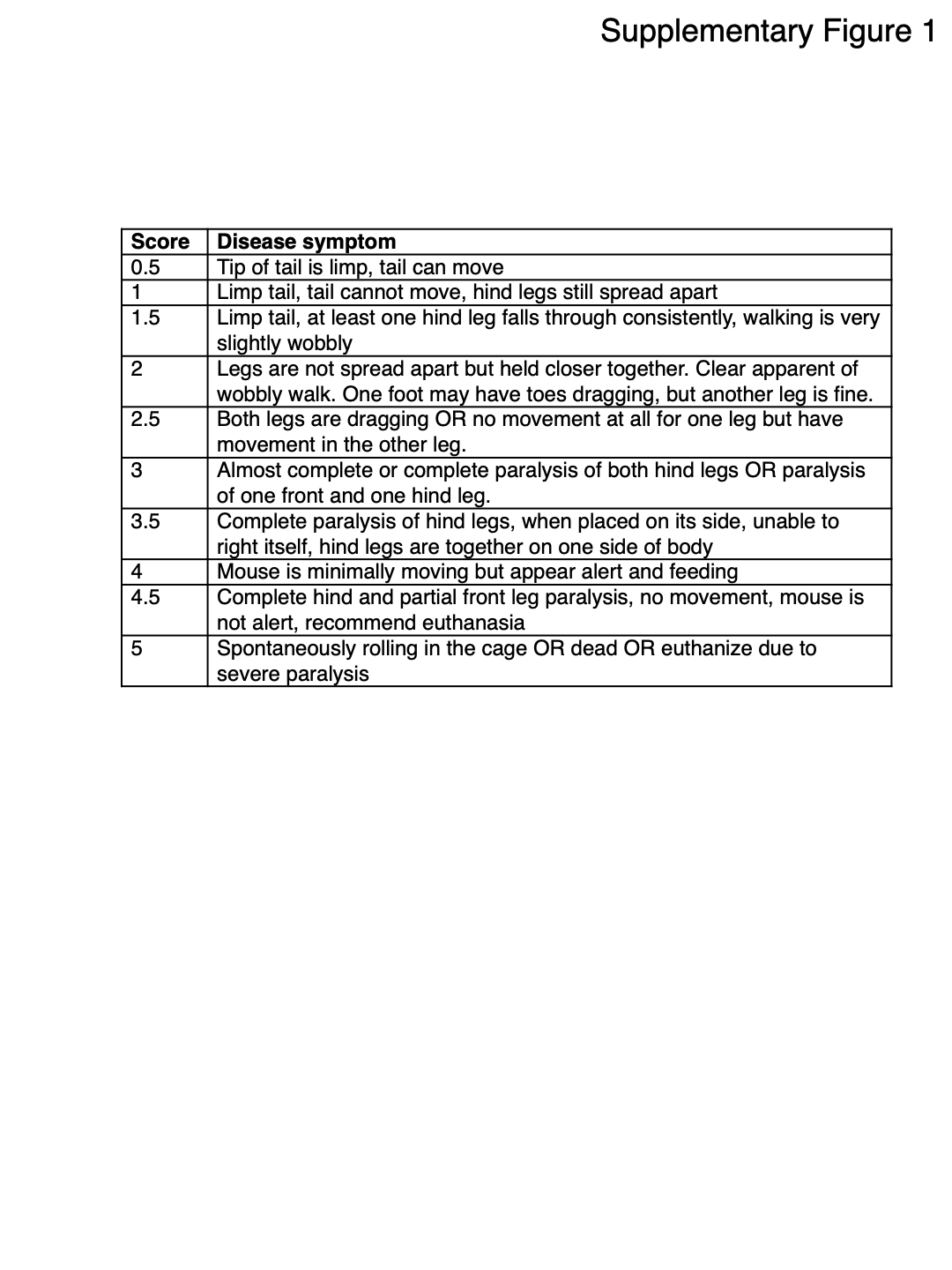

Supplement: Supplementary Figure 1 — Disease symptoms and criteria for determining the clinical scores of EAE. [file Image_1.tiff]

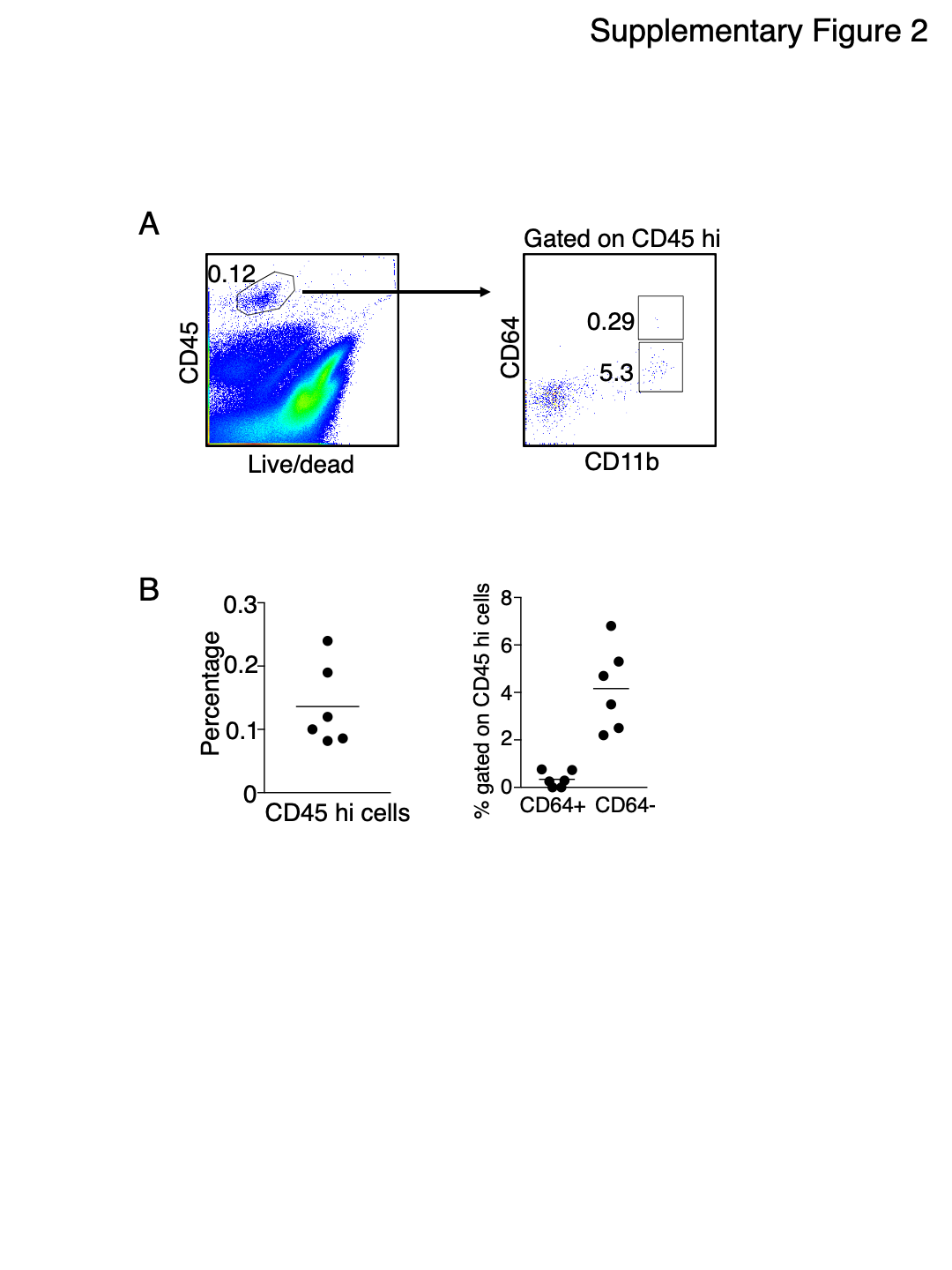

Supplement: Supplementary Figure 2 — CD64+ monocytes are not detected in mice without EAE induction. (A,B) Spinal cords from the naïve C57BL/6J mice were removed. Percentage of the CD45+ CD11b+ CD64+ and the CD45+ CD11b+ CD64lo/− cells was determined. (A) Shown are representative plots from two independent experiments with a total of six mice. (B) Data from individual animals are shown. [file Image_2.tiff]

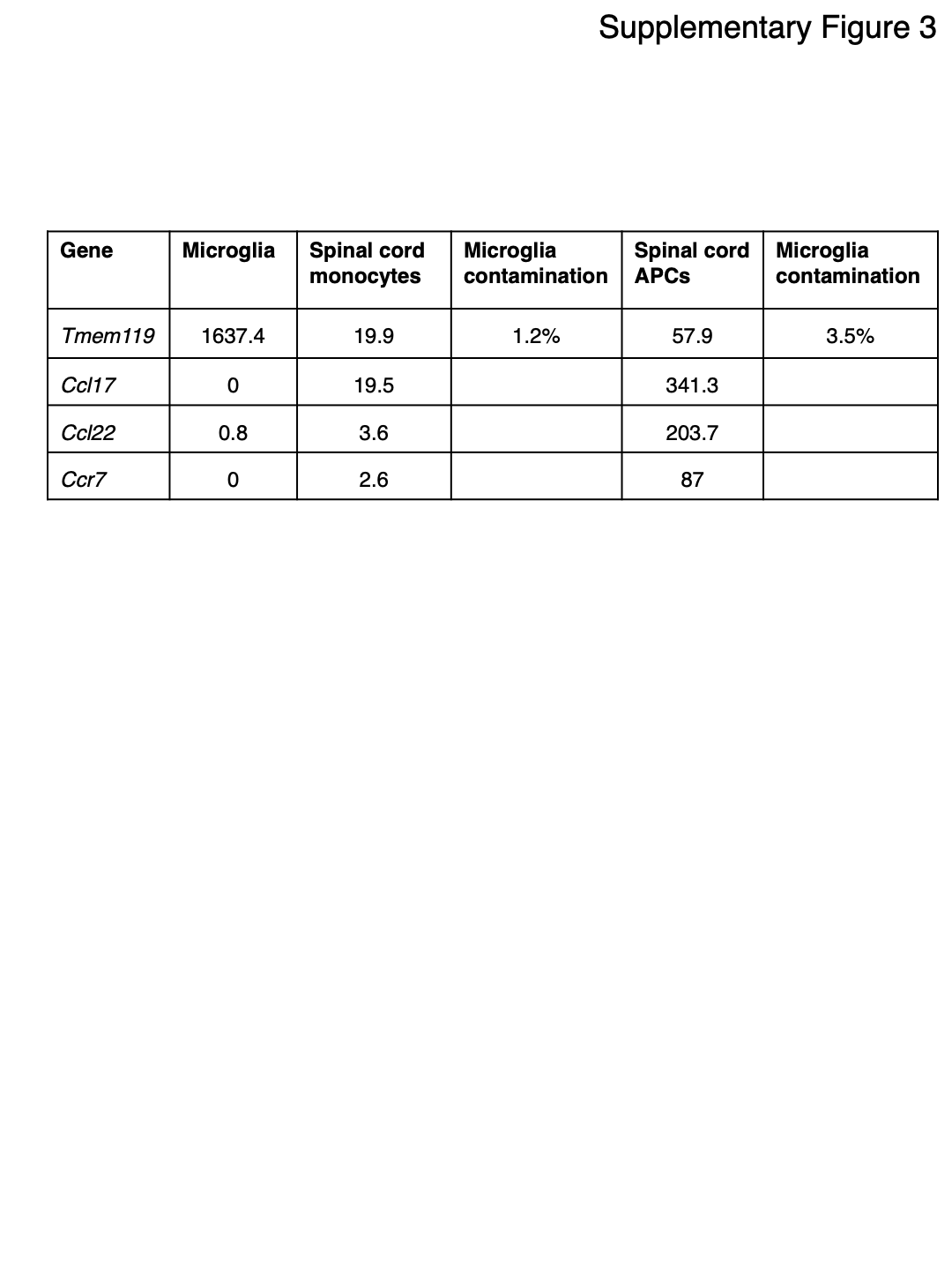

Supplement: Supplementary Figure 3 — Comparison of the expression levels of Tmem119, Ccl17, Ccl22, and Ccr7 in the monocytes and the monocyte-derived APCs to microglia. Shown are TPM values normalized with the expression of Actb across datasets. Percentage of microglia contamination is calculated by the expression of Tmem119 in the microglia over spinal cord monocytes or spinal cord APCs. Gene expression data of microglia were obtained from results published previously (31). [file Image_3.tiff]
